# A multi‐omics‐based investigation of the immunological and prognostic impact of necroptosis‐related genes in patients with hepatocellular carcinoma

**DOI:** 10.1002/jcla.24346

**Published:** 2022-03-15

**Authors:** Hang Yang, QiNian Jiang

**Affiliations:** ^1^ 74628 Guizhou Medical University Guiyang China

**Keywords:** biomarkers, drug screening, hepatocellular carcinoma, necroptosis, prognosis

## Abstract

**Background:**

Hepatocellular carcinoma (HCC) is the most common histological subtype of liver cancer and the third leading cause of death from cancer globally. Recent studies suggested cell death is also a key regulator of tumour progression. The purpose of this study was to generate a new predictive signature for HCC patients based on a complete analysis of necroptosis‐associated genes.

**Methods:**

We extracted the mRNA expression profiles of HCC patients from the TCGA and ICGC databases and their clinical data. In addition, we used the IMvigor210 cohort to validate our model molecule's ability to predict the effect of immunotherapy. In the TCGA cohort, a seven‐gene risk‐prognostic model was constructed using univariate cox‐Lasoo regression. External validation was conducted using the ICGC cohort. The ssGSEA algorithm is used to determine the degree of immune function response. The CMAP databases are used for chemotherapy drug analysis and screening for drugs that reduce the expression of high‐risk genes. The cbioportal database was used to explore mutations in model genes.

**Results:**

Survival analysis shows shorter survival for high‐risk patients. Immune function analysis revealed significant differences in the activity of immune pathways between risk subgroups. Varied risk scores result in dramatically diverse immune infiltration and tumour growth, as well as significantly different chemotherapeutic sensitivity. In addition, Apigenin and LY‐294002 reduced the expression of high‐risk genes, while Arecoline had the opposite effect. In the immunotherapy IMvigor210 cohort, risk scores were significantly different between the objective responder and non‐responder groups. By comparing the models constructed with published literature, it is suggested that our model has better predictive power.

**Conclusions:**

We created a new prognostic signature of necroptosis‐related genes that can be used as potential prognostic biomarkers to guide effective personalized therapy for hepatocellular carcinoma patients.

## Graphical abstract

A prognostic model for necroptosis‐related genes was first constructed by Lasso regression, followed by validation at the single cell and protein levels. This is followed by clinical correlation analysis to verify whether the model can be used independently as a prognostic factor. At the end, chemotherapy drug sensitivity analyses are performed and drugs that reduce the risk to patients are identified.

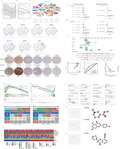

## 1 INTRODUCTION

Ninety percent of primary liver cancers are caused by hepatocellular carcinoma (HCC), the third most common cause of cancer‐related death globally. According to the American Cancer Society, in 2021 liver cancer is expected to cause approximately 30,230 American deaths.^1^ Genetics, epigenetic alterations, chronic hepatitis B, are the main risk factors for hepatocellular carcinoma. Hepatocellular carcinoma has a poor prognosis because of its propensity for recurrence and dissemination. Genetics, epigenetic alterations, chronic hepatitis B, are the main risk factors for hepatocellular carcinoma. Hepatocellular carcinoma has a poor prognosis because of its propensity for recurrence and dissemination.

Immune checkpoint inhibitors (ICIs) have become an effective therapy option for patients with advanced HCC in recent years due to their increased clinical use. Clinical agents for treating HCC with checkpoint inhibitors include anti‐CTLA‐4 and anti‐PD‐1 drugs. Anti‐PD‐1 drugs have demonstrated significant effects in improving tumour response and patient survival.^2^ However, immune checkpoint inhibitors (ICIs) only benefit one‐third of cancer patients and have significant limitations.

The majority of cancers are very resistant to apoptosis, and induced cell death processes can be a brand new cancer treatment, and numerous recent research has established a link between various cell death mechanisms and anticancer immunity. Recent studies have revealed that pyroptosis and ferroptosis combined with immune cell infiltration can affect the progression of different cancers and have developed some novel prognostic molecules.^3^, ^4^ However, few studies have explored how necroptosis affects the progression of hepatocellular carcinoma and the immune infiltration of hepatocellular carcinoma cells.

Necroptosis occurs downstream of PRK1 and RIPK3, which form oligomeric complexes known as necrosomes.^5^ MLKL mediates the release of cell contents by necrosome‐promoted cell swelling and plasma membrane collapse, resulting in the spillage of intracellular organelles and biomolecules into the extracellular environment. Necroptosis has been shown to inhibit tumour progression, but it can also promote cancer metastasis and immunosuppression by eliciting an inflammatory response.^6^, ^7^ However, the mechanism of necroptosis's role in hepatocellular carcinoma remains unknown, so this study used bioinformatics to investigate the prognosis of necroptosis‐related molecules as well as the immunological role.

## 2 MATERIALS AND METHODS

### 2.1 Data acquisition and collation

The TCGA database (https://portal.gdc.cancer.gov/) was utilized to download transcriptome datasets (FPKM) and clinical information for 424 samples of hepatocellular carcinoma (50 normal and 374 tumour samples). As a training set, we use the TCGA cohort. We also downloaded the Japanese cohort's transcriptome and clinical information gleaned from the ICGC database (https://dcc.icgc.org/projects/LIRI‐JP) and obtained 231 samples with survival time, outcome and pathological stage as the validation cohort by collating and merging the transcriptome and clinical data (Appendix S1 and S2). In addition, we gathered 62 genes related to the necroptosis pathway for inclusion in this study by searching the literature. In addition, the immunotherapy IMvigor210 cohort was derived from the literature.^8^ Because TCGA and ICGC are open source databases, there are no ethical concerns or conflicts of interest.

### 2.2 Differentially expressed necroptosis‐related genes with prognostic effects

We extracted the expression of 62 genes associated with necroptosis from the TCGA transcriptome as a new matrix for subsequent analysis, and identified 42 genes differentially expressed between the tumour and normal groups using the ‘limma’ package^9^ [logFold change >1, false discovery rate <0.05]. Screening of necroptosis genes associated with prognosis by univariate COX analysis (Appendix S1 and S2). Prognosis‐related differentially expressed genes were identified by taking intersections using the Venn package. (PR‐DEGs).

### 2.3 Gene ontology and KEGG analysis of differentially expressed genes

We used ‘clusterProfiler’ package^10^, ^11^ to transform IDs of DEGs and performed gene ontology and Kyoto Encyclopedia of Genes and Genomes analysis, and ‘ggplot2’, ‘enrichplot’ packages to visualize the obtained data.

### 2.4 Development of the necroptosis‐related gene prognostic model

Screening of PR‐DEGs by the LASSO algorithm of the ‘glmnet’ R package to identify genes for model building. Seven genes' expression levels and regression coefficients were used to determine the risk scores for HCC patients (Table 1). The equation employed was:

$$\begin{array}{cc} \text{Riskscore} & =0.10539\times \text{TRAF}2+\text{PGAM}5\times 0.38577+\text{ATG16L1}\times 0.10023\\ & +\text{CARD}9\times 0.33098+\text{PCYT1A}\times 0.16166+\text{TLR2}\times 0.02069\\ & +\text{PARP}2\times 0.19336 \end{array}$$

**TABLE 1:** Coefficients of prognostic genes obtained based on L asso algorithm

Gene	Coef
TRAF2	0.105398580022597
PGAM5	0.385771773843994
ATG16L1	0.100232667095849
CARD9	0.330982332251438
PCYT1A	0.161666550305379
TLR2	0.020691625065588
PARP2	0.193368483419255

Patients were classified as high‐ or low‐risk depending on the TCGA training set's median risk scores. The expression matrix of the ICGC validation set is also $\log_{2}\;\left(X+1\right)$‐normalized, and the TCGA training set's median risk score is used as the criteria for grouping the ICGC cohort. (Appendix S1 and S2). To determine the predictive accuracy of the risk scores, ROC curves were constructed using the ‘Survival’ and ‘Time ROC’ packages. The ‘Rtsne’ and ‘ggplot2’ packages were used for PCA and t‐SNE analysis and visualization to explore whether our risk model can better distinguish between different patients. We collected immunohistochemical profiles of the corresponding necroptosis‐related genes through the Human Protein Atlas^12^, ^13^, ^14^ database(https://www.proteinatlas.org/) to identify trends in differential gene expression in different tissues. The Single Cell Expression Atlas (SCEA) database project was^15^ used to explore the expression of key genes in hepatocellular carcinoma at the single‐cell level. In addition, the immunotherapy IMvigor210 cohort was used to validate the model's ability to predict immunotherapy.

### 2.5 Risk prognostic model independent prognostic analysis

We analysed whether a risk‐prognosis model for two independent cohorts could be distinguished from traditional clinicopathological characteristics as an independent prognostic indicator for patients by univariate Cox and multivariate Cox . Based on the results of multivariate COX analysis we built the Nomogram (Appendix S1 and S2) and then labelled a patient's score information for clinical use. Calibration curves occur at 1, 2 and 3 years. Multi‐indicator ROC curves and decision curve analysis (DCA) curves and calibration curves are used to assess the accuracy of the Nomogram.

### 2.6 Identifying differences in gene enrichment and pathological features between risk subgroups

We analysed the differences in the enrichment pathways between the different risk subgroups by the gene set enrichment analysis (GSEA) algorithm.^16^ Patient expression data were combined with clinical data to observe differences in clinical characteristics between the high‐ and low‐risk groups of the two cohorts of TCGA and ICGC by chi‐square test(Appendix S1 and S2).

### 2.7 Differential analysis of immune cells and function in different risk subgroups

We obtained the immune score using the ssGSEA algorithm in the GSVA package^17^ and visualized the differences in immune cells and function in different risk subgroups by plotting box plots. We included 19 immune checkpoint‐related genes, extracted significantly differentially expressed between risk subgroups using the Wilcox test, and visualized gene expression differences using box plots(Appendix S1 and S2).

### 2.8 Drug sensitivity analysis and CMAP drug screening

The drug sensitivity files were downloaded by accessing the NCI‐60 database through CellMine (https://discover.nci.nih.gov/cellminer), and Pearson correlation analysis was used to investigate the relationship between model gene expression and drug sensitivity to correlate the efficacy of FDA‐approved drugs (Table 2) (Appendix S1 and S2).

**TABLE 2:** CMAP database top 10 drug screening results

Rank	Cmap Name	Mean	N	Enrichment	*p* value	Specificity	Percent non‐null
1	Verteporfin	−0.896	3	−0.993	0	0	100
2	Apigenin	−0.834	4	−0.952	0	0	100
3	Tanespimycin	−0.524	62	−0.44	0	0.0924	79
4	Trichostatin A	−0.397	182	−0.267	0	0.6238	71
5	Phenoxybenzamine	−0.838	4	−0.932	0.00002	0.0091	100
6	Adiphenine	0.735	5	0.925	0.00002	0	100
7	LY−294002	−0.441	61	−0.325	0.00002	0.2883	73
8	Biperiden	0.702	5	0.852	0.00018	0.0061	100
9	Alprostadil	0.443	7	0.739	0.00022	0	71
10	Pheneticillin	0.644	4	0.877	0.00034	0	100

We obtained different genes by differential analysis of high‐risk and low‐risk subgroups. In order to reduce the risk of patients and improve survival, we performed drug screening through the CMAP database (https:/portals.broadinstitute.org/cmap/) and searched the PubChem website (https:/pubchem.ncbi.nlm.nih.gov/) for two‐dimensional and three‐dimensional structural formulas of drugs(Appendix S1 and S2).

### 2.9 Mutation analysis of model genes

We visualized mutations in different risk subgroups using the maftools package, analysed the relationship between tumour mutation burden (TMB) and risk score, and examined mutations in model genes with corresponding amino acid structural domain mutations using the cbioportal (http://www.cbioportal.org/) database.

### 2.10 Comparison between prognostic risk models

We collected literature on hepatocellular carcinoma,^18^, ^19^, ^20^ extracted the genes they used to construct predictive models and performed survival curve and ROC curve analysis to compare the predictive power of our constructed models.

### 2.11 Statistical analysis

All statistical analyses including Kaplan–Meier survival analysis and univariate multivariate COX analysis were done in R language version 4.1.1, and *p* values <0.05 were considered statistically significant in different comparisons.

## 3 RESULTS

### 3.1 Identification of genes differentially expressed in different tissues in relation to prognosis

**FIGURE 1:**
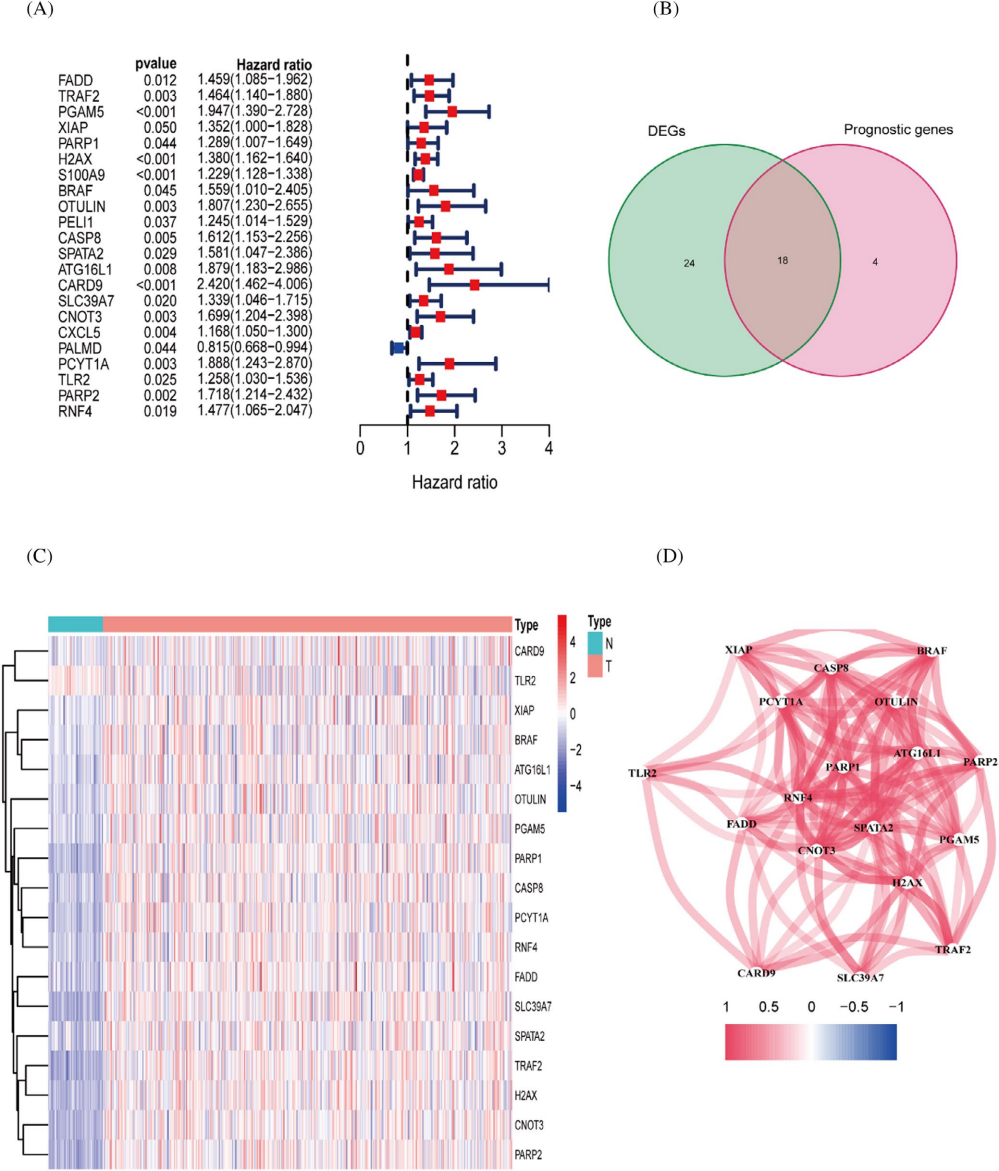
Identification of genes differentially expressed in different tissues in relation to prognosis. (A) Univariate COX analysis of prognosis‐related genes. (B) The intersection portion of the Wayne plot shows 18 differentially expressed genes associated with prognosis. (C) Heat map showing differential expression of genes. (D) Correlation graph between genes

Through univariate COX analysis, we identified 22 genes with prognostic significance. (Figure 1A). 18 PR‐DEGs were obtained by taking intersections of differentially expressed genes with prognosis‐related genes (Figure 1B). Heatmap demonstrates the difference in expression of 18 PR‐DEGs in tumour and non‐tumour tissues (Figure 1C). Correlation diagram showing the different associations between the 18 PR‐DEGs (Figure 1D).

### 3.2 Gene ontology and KEGG analysis of DEGs

**FIGURE 2:**
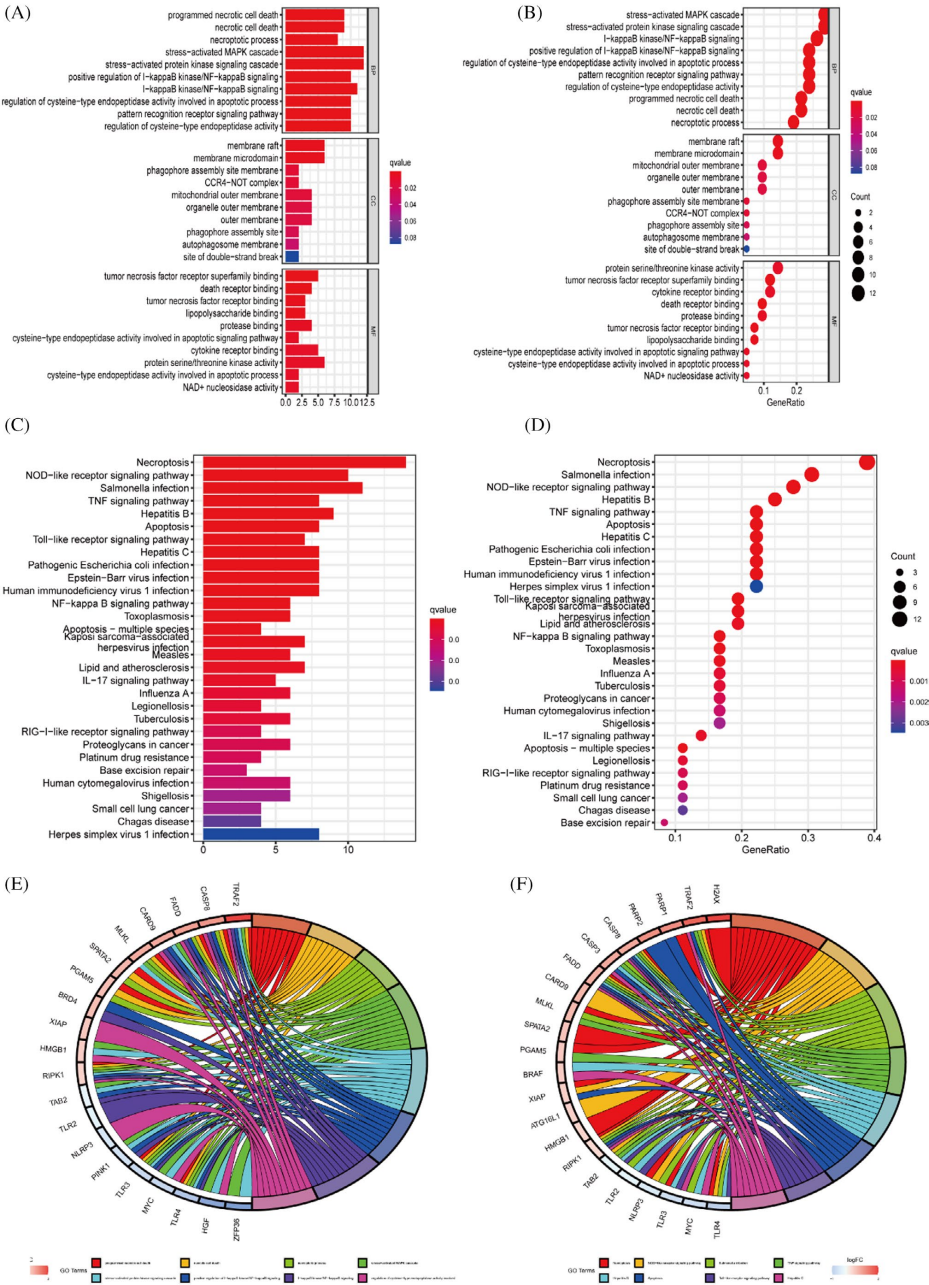
Gene enrichment and pathway analysis. (A,B) Gene ontology enrichment analysis. (C,D) KEGG analysis with these differential genes was enriched in Necroptosis, TNF signalling pathway. (E,F) Circle diagram for GO and KEGG analysis

We have used gene ontology to analyse the three components of gene involvement: cellular components, molecular functions and biological processes. We found that DEGs are mainly enriched in the biological process of stress‐activated MAPK cascade at the BP level. In terms of cellular components, they mainly constitute membrane rafts, which are microstructural domains enriched in cholesterol‐saturated lipids (e.g. sphingolipids) but insoluble in Triton X‐100. In addition, for molecular functions DEGs are concentrated in protein serine/threonine kinase activity(Figure 2A,B,E). In addition, KEGG analysis revealed that DEGs were mainly enriched in Necroptosis and NOD‐like receptor signalling pathways(Figure 2C,D,F).

### 3.3 Risk prognostic modelling and external cohort validation

**FIGURE 3:**
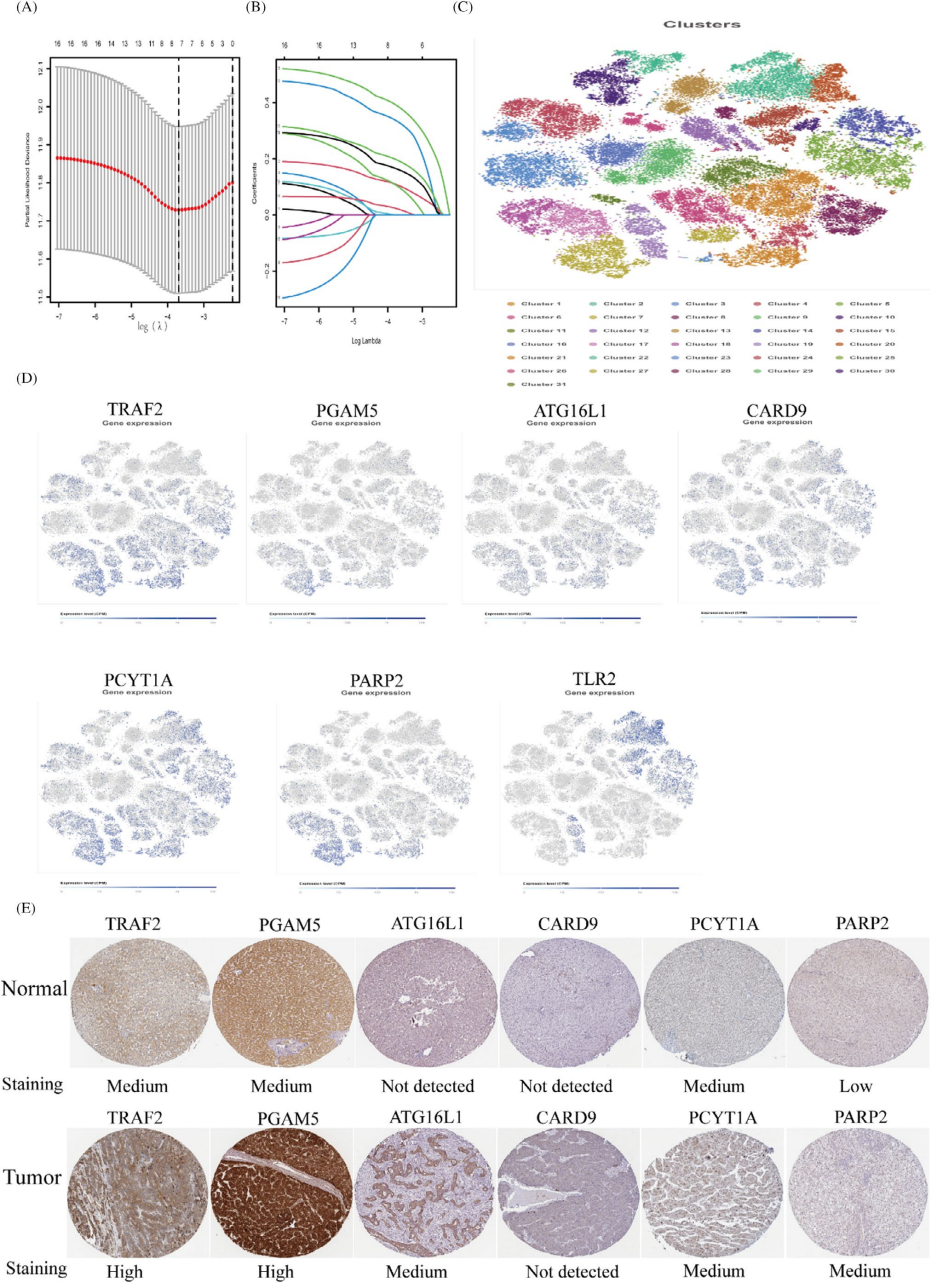
Participate in the screening and identification of construct model genes. (A,B) LASSO coefficient plots and 10‐fold cross‐validation plots for seven model genes. (C,D) In the SCEA database, all hepatocellular carcinoma monocytes were clustered into 31 subgroups after setting the appropriate parameters. Most of the model genes can be used as marker genes for group 7. (E) Differences in expression trends of model genes in different tissues in the HPA database

Seven genes involved in the construction of the model were identified by univariate cox and lasso analysis. The sum of the coefficients of the seven genes and the product of their respective expressions was the patient's risk score (Figure 3A,B; Table 1). The single‐cell clustering plots demonstrate the differences in expression of different model genes in different clusters, and most genes are highly expressed in cluster 7 (Figure 3C,D). Immunohistochemistry map of the HPA database illustrating gene expression trends in various tissues. Significantly high expression of TRAF2, PGAM5 and ATG1621 in the tumour group was an unfavourable prognostic factor. PCYTIA and CADR9 are not differentially expressed. The results obtained from the HPA database KM survival analysis and our analysis were consistent as an unfavourable prognostic factor (Figure 3E).

**FIGURE 4:**
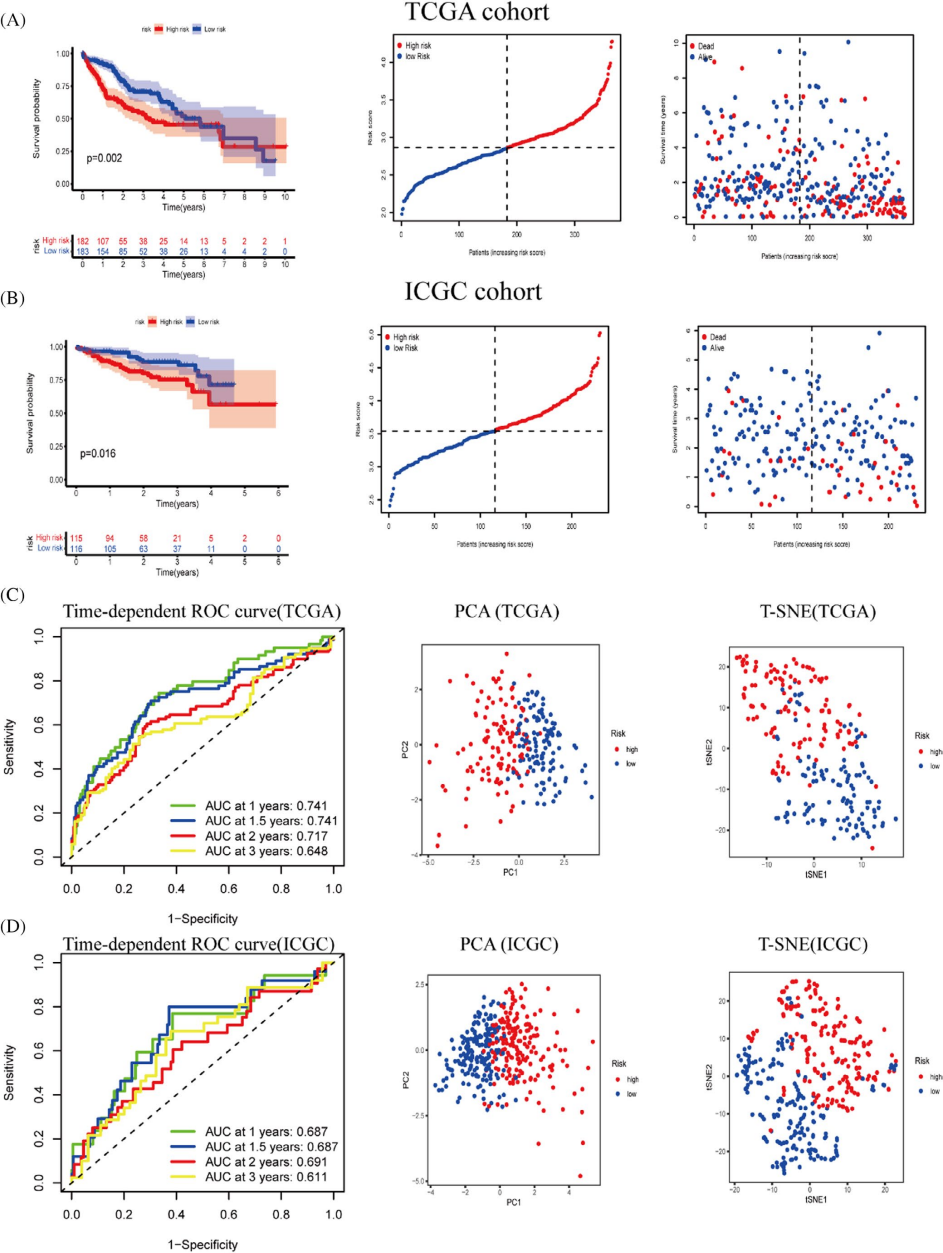
Construction of a risk prognostic model with external cohort validation. (A) Construction of a risk‐prognosis model using the TCGA cohort as the training set. (B) ICGC cohort for validating the prognostic value of risk prognostic models. (C,D) Analyses of the receiver operating characteristic curves for risk scores, and PCA and T‐SNE analysis revealed that our model was more capable of discriminating between patients with varying levels of risk

The Kaplan–Meier survival curve results showed that the survival rate was significantly lower in the high‐risk group of the TCGA cohort (*p* = 0.002) and ICGC cohort (*p* = 0.016) (Figure 4A,B). The area under the ROC curve for the TCGA cohort at 1, 2 and 3 years was 0.741, 0.717 and 0.648. In the ICGC cohort, the AUCs were 0.687, 0.691, 0.611. The AUC values of the ROC curves for the two cohorts revealed the predictive power of our constructed model in different datasets. PCA and t‐SNE analyses demonstrate that risk models can more accurately distinguish between patients with varying degrees of risk (Figure 4C,D).

### 3.4 Independent prognostic analysis of risk prognostic models and comparison with multiple indicators

**FIGURE 5:**
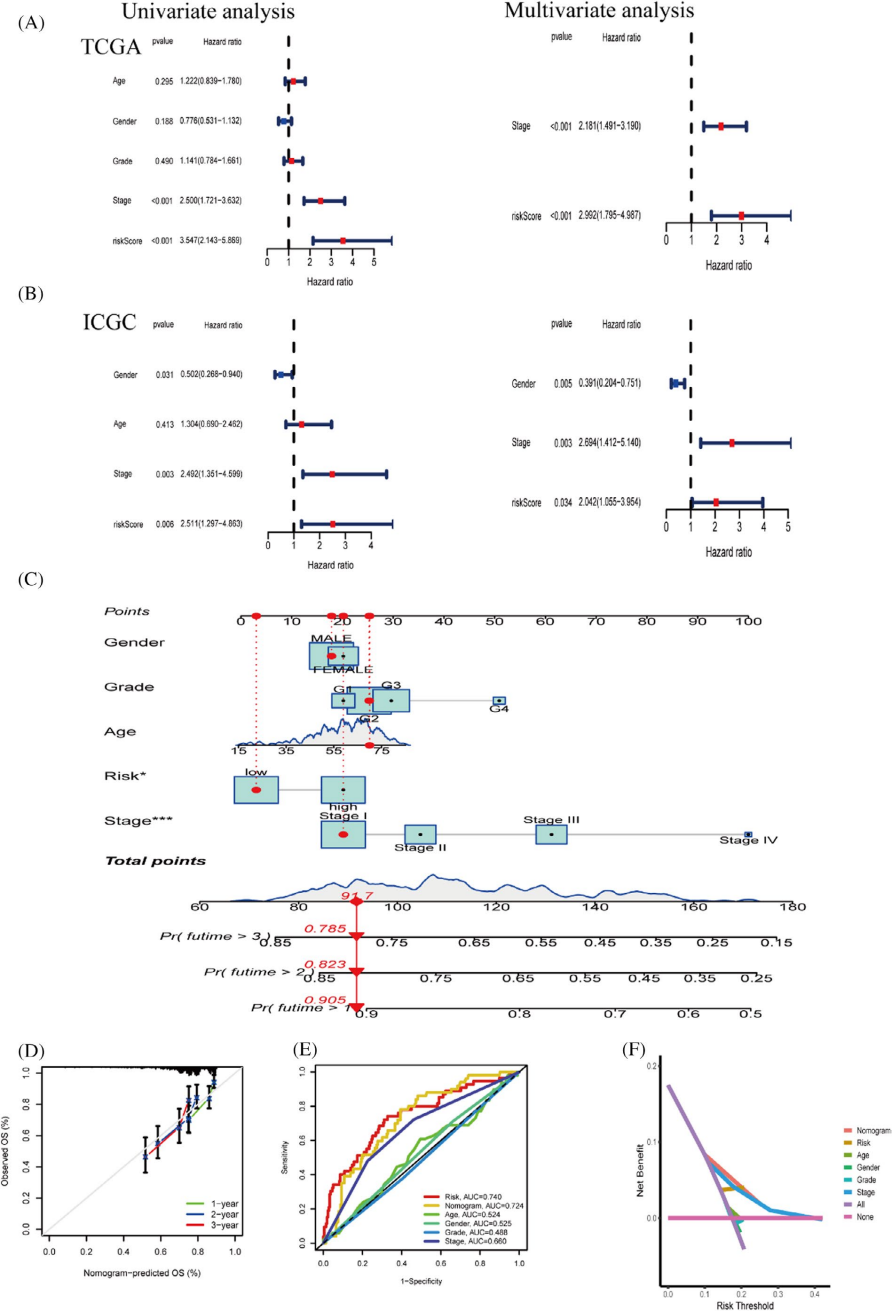
Independent prognostic analysis of risk prognostic models. (A,B) COX analysis on a univariate and multivariate basis in the TCGA and ICGC cohorts (C) Predictive nomogram for predicting patients at 1, 3 and 5 years. (D) Calibration curve showing the stability of the nomogram. (E) Multi‐Indicator ROC Curve. (F) Decision curve analysis(DCA)

In both the TCGA and ICGC cohorts, univariate and multivariate cox analyses revealed that stage of risk score and pathological characteristics were significant predictors (Figure 5A,B). In addition, to better utilize patient information and reduce the errors associated with the prognosis of individual indicators, we combined the patient's stage, gender, grade, age, risk information to develop a nomogram. We randomly labelled the corresponding clinical information and the patient's total score on the graph (Figure 5C). The calibration curve shows the stability of the Nomogram (Figure 5D). The ROC curve demonstrates the specificity and sensitivity of the predictive ability of each prognostic indicator (Figure 5E). The DCA curve shows the best predictive ability of our constructed Nomogram model (Figure 5F).

### 3.5 Differences in clinical characteristics and gene enrichment among different risk subgroups

**FIGURE 6:**
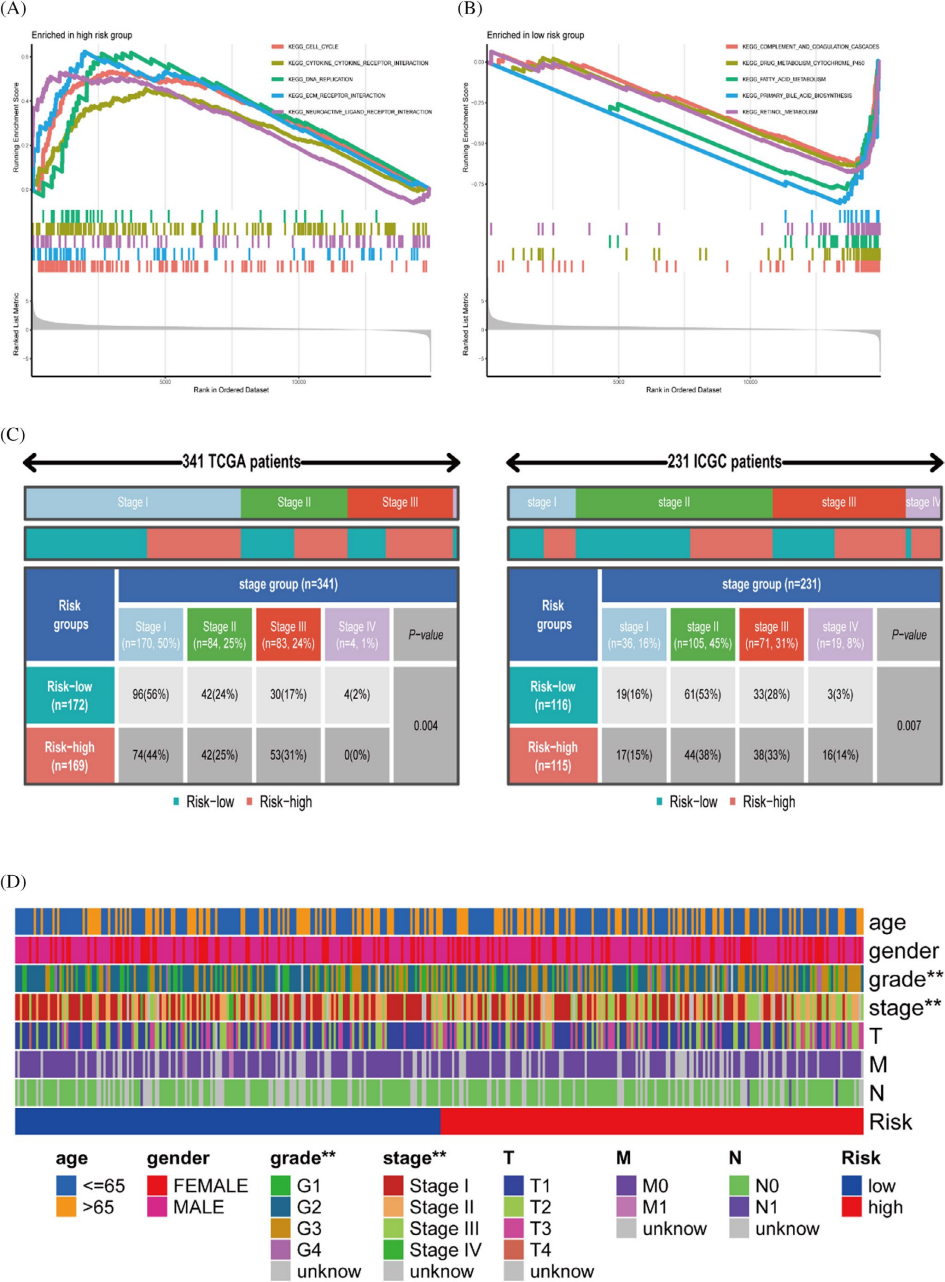
Differences in clinical characteristics and gene enrichment among different risk subgroups. (A, B) GSEA enrichment analysis results. (C) Analysis of the relationship between risk and stage in TCGA cohort and ICGC cohort. (D) Significantly different clinical characteristics in different risk subgroups

Gene set enrichment analysis results found that the high‐risk group was associated with cell cycle, base excision repair and cytokine receptor interaction pathway (Figure 6A). The low‐risk group was enriched in drug metabolism cytochrome ‐P450 (Figure 6B). The rectangular plots show the differences between the clinicopathological characteristics stage between different risk subgroups in two independent cohorts. The stage was significantly different between different risk subgroups in the TCGA (*p* = 0.004) and similarly in the ICGC (*p* = 0.007), and in addition, we explored the different clinicopathological characteristics between different risk subgroups, and we marked the indicators with significant differences (Figure 6C,D).

### 3.6 Differences in immune cells and function in different risk subgroups

**FIGURE 7:**
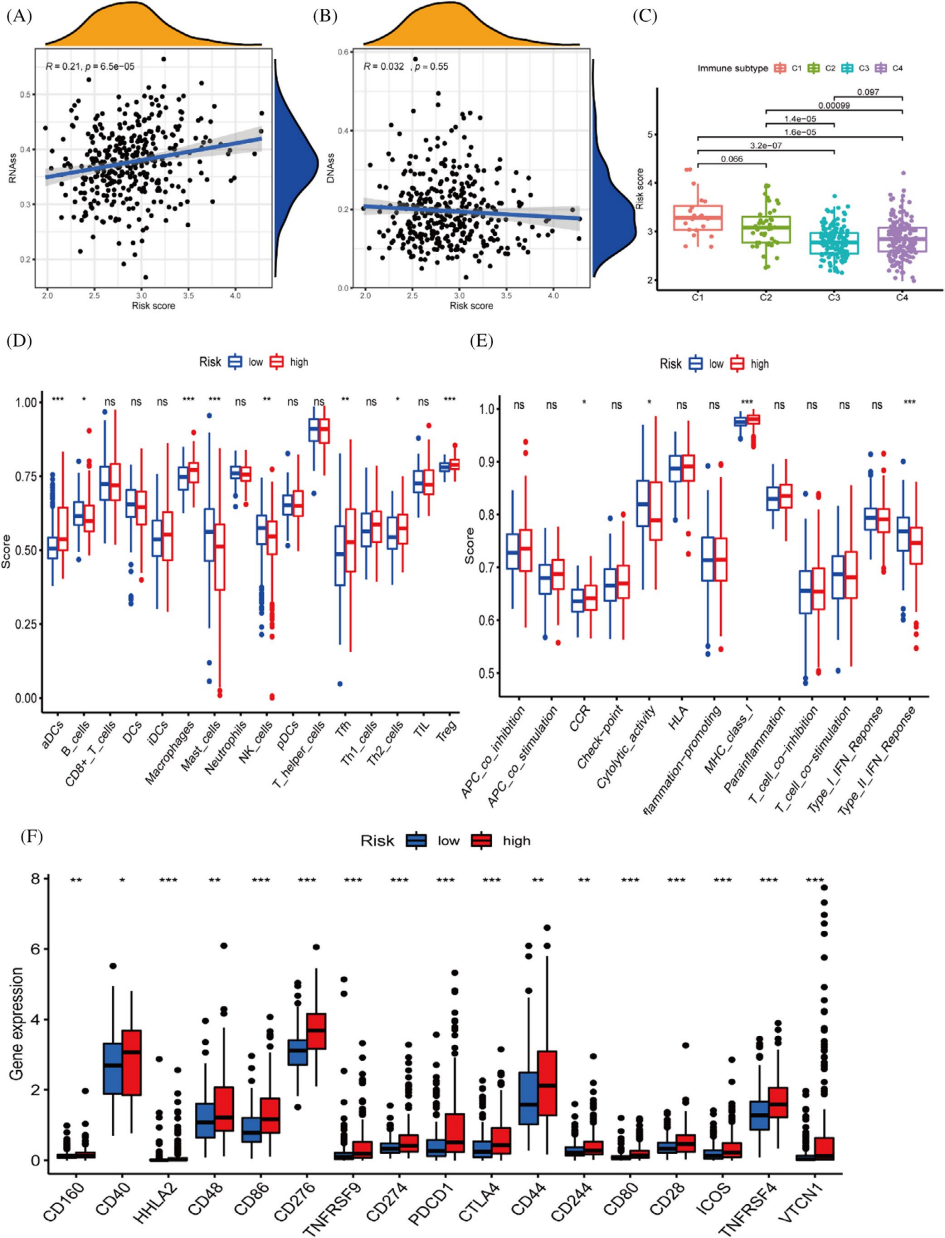
Immune cell and functional differential analysis based on different risk subgroups. (A, B) Tumour stem cell correlation analysis. (C) Differences in RiskScore between different immunophenotypes. (D, E) Immune function analysis of the TCGA cohort. (F) Immune checkpoints express differently in different risk subgroups

Tumour stem cell correlation analysis was performed by mRNA expression and DNA methylation data. There is a statistically substantial correlation between risk scores and RNA stemness scores (RNAss), but risk score was not correlated with DNA stemness score (DNAss) (*p* = 0.55) (Figure 7A,B). We aimed to discuss whether the risk groupings we constructed differed between subtypes, and the results of the analysis showed significant differences between subtypes except for C3 and C4 where the risk scores did not differ significantly (*p* = 0.097) (Figure 7C). Using the SSGSEA algorithm, we obtained immune scores for each patient and explored the differences in immune function based on the previous risk subgroups. aDCS, macrophages and treg immune cells were significantly more infiltrated in the high‐risk group than in the low‐risk group (Figure 7D). In addition, the Type II IFN Response cytolytic activity functional pathway was more active and significantly different in the low‐risk group than in the high‐risk group (Figure 7E). We included 19 immune checkpoint‐associated genes to explore the differences between high‐ and low‐risk subgroups (Figure 7F).

### 3.7 Drug sensitivity analysis and screening of drugs

**FIGURE 8:**
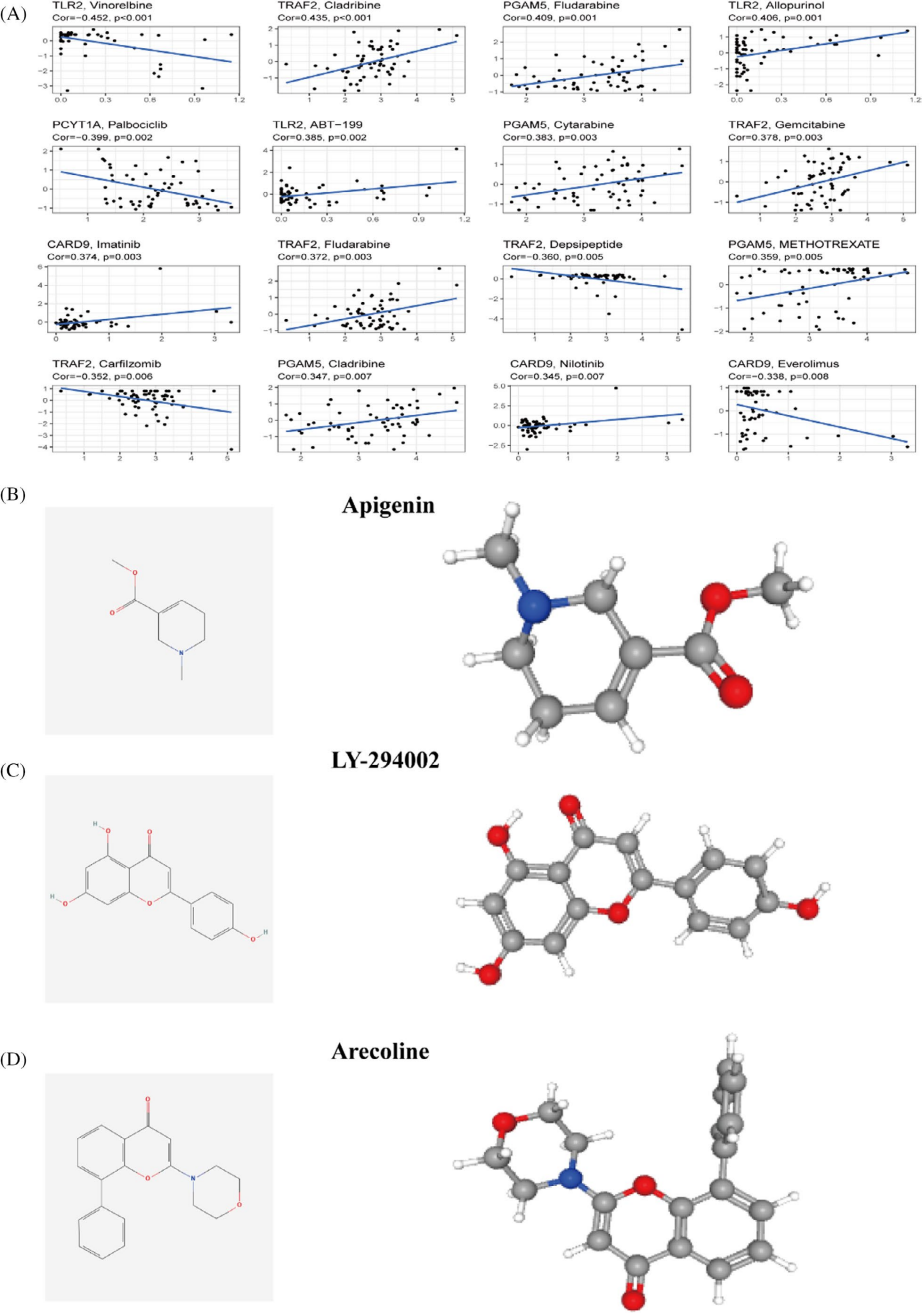
Drug sensitivity analysis and drug screening. (A) Correlation analysis of chemotherapeutic drug sensitivity and different gene expressions.| Identification of drugs that reduce and promote the expression of high‐risk genes. (B) Apigenin. (C) LY‐294002. (D) Arecoline

We investigated the expression of model genes involved in risk prognosis in the NCI‐60 cell line by looking at which model genes affect the sensitivity of drugs through sensitivity analysis. The correlation graphs show these results, where cor values greater than 0 and *p* values <0.05 indicate that higher gene expression is more sensitive to the drug and vice versa. For example as the expression of the PGAM5 gene increases, the more sensitive the cells are to Cytarabine, the better the treatment effect (Figure 8A). In the above GSEA analysis study, the low‐risk group was enriched in the process of drug metabolism P450. We conducted a differential analysis based on risk groups to identify high‐risk genes and conducted drug screening through the CMAP database to select drugs to reduce patient risk and improve patient survival. Enrichment score <0 and *p*‐value <0.05 were considered drugs that could inhibit the expression of high‐risk genes. We searched for the two‐dimensional and three‐dimensional structures of related drugs through the Pubchem website to help us better understand the drugs (Figure 8B‐D).

### 3.8 Model gene mutation analysis

**FIGURE 9:**
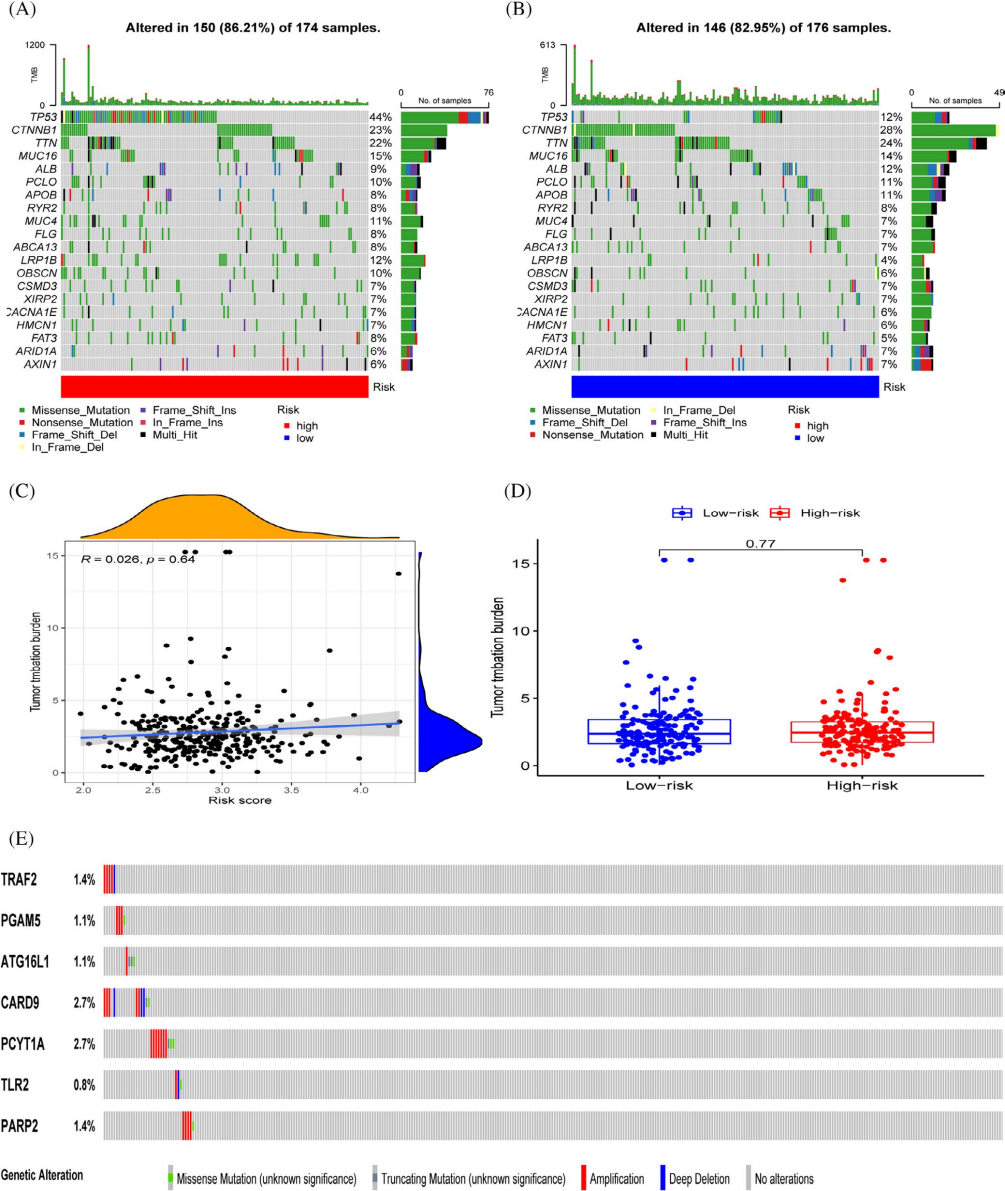
Genetic mutation landscape. (A‐B) TP53, CTTNB1 and TTN had the highest number of mutations in both groups. (C,D) Relationship between tumour mutation burden and risk score. (E) Mutation of model genes between samples

Waterfall plots show the mutations in different risk subgroups, where TP53, CTNNB1, TTN gene mutations are more frequent (Figure 9A,B). There was no significant correlation between tumour mutation burden and risk score, and the difference in TMB was not significant in different risk subgroups (Figure 9C,D). The cbioportal database demonstrates mutations in model genes in TCGA samples (Figure 9E), and also demonstrates mutations in amino acid structural domains, but most genes are not significantly mutated (Figure S1).

### 3.9 Comparison between different risk prognostic models

**FIGURE 10:**
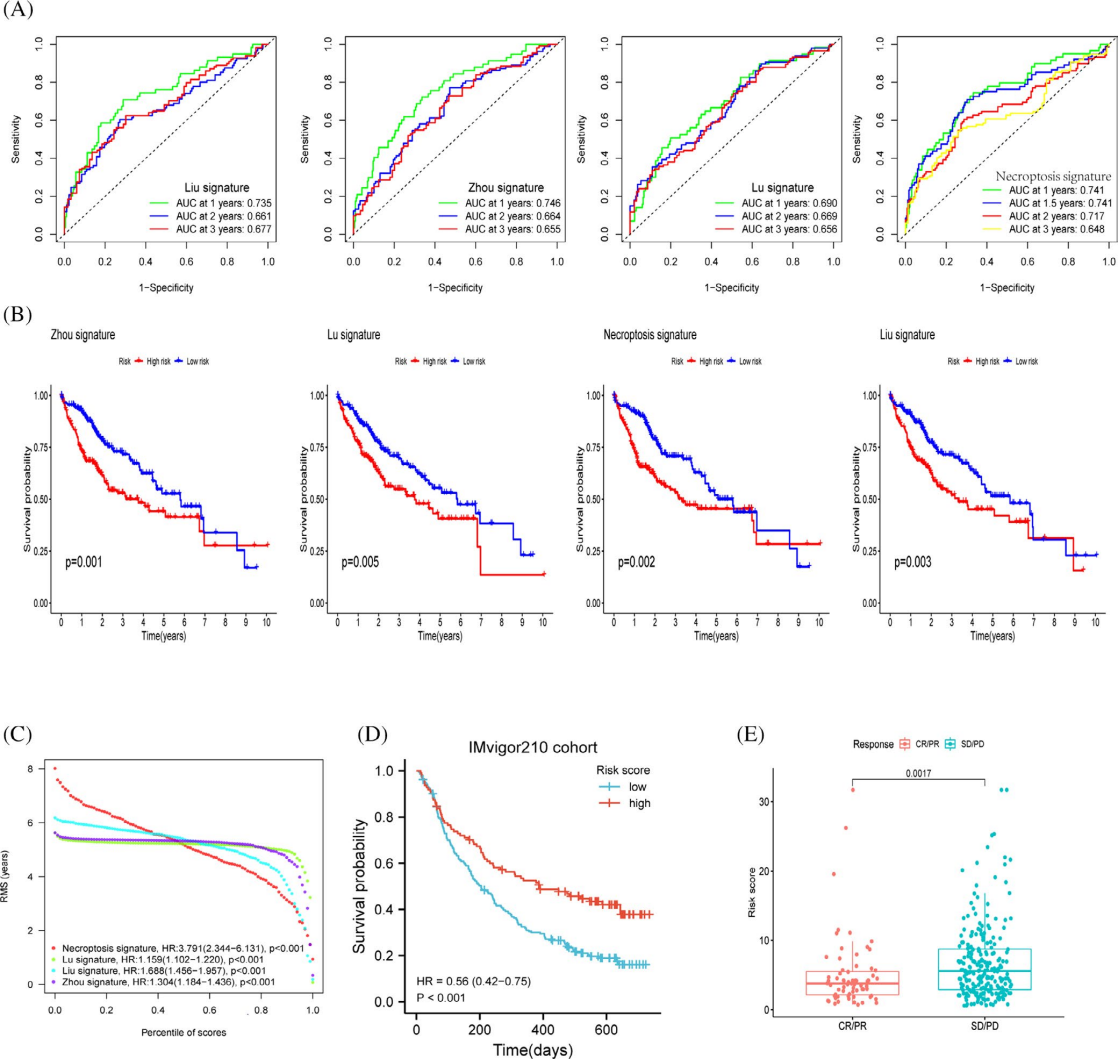
Comparison and analysis between models. (A‐C) Comparison of the three authors with our model, ROC curve and KM curve demonstrates the predictive power metrics of different models. (D) Survival curve analysis of the immunotherapy cohort. (E) Variability between risk scores among different response groups

The ROC curve results suggest that our model has better predictive power, especially in predicting the survival of patients in the second year (Figure 10A), as demonstrated by the results of the survival curve when compared with the prognostic models constructed by the other three authors (Figure 10B,C). In addition, using the IMvigor210 cohort, we found that our model could also predict to some extent the effect of immunotherapy in patients, with statistically significant differences in risk scores between the objective responder and non‐responder groups (Figure 10D,E).

## 4 DISCUSSION

Identification of necrotizing apoptosis often requires multiple approaches because no specific molecular markers have been identified to date for detecting necroptosis. Additionally, the impact of necrosis on tumour progression remains unknown, although some reports indicate that necrosis may have an anti‐tumour effect in cancer. However, necrosis has been shown to promote tumorigenesis and metastasis by inducing an inflammatory response, as Liu et al.^21^ reported that it has been reported that silencing RIPK1 and RIPK3 in cancer cells decreases the pathogenic capacity of cancer cell lines and increases their sensitivity to chemotherapy. Seifert et al.^6^ demonstrated that silencing RIPK1 in mice slowed tumour progression in animal experiments, suggesting that necroptosis‐related factors promote cancer development.

In this study, we collected previous literature on necroptosis and included 62 genes related to necroptosis for bioinformatic analysis, and we identified 18 molecules with prognostic value. We observed that the HR values of these genes were all greater than 1 as risk factors, and the results of immunohistochemical profiles support this conclusion. Upregulation of expression of genes involved in model constructs promotes cancer progression, implying that trends in the expression of necroptosis molecules are inconsistent across cancer types. We constructed a risk‐prognosis model based on the lasso algorithm, and all genes involved in the construction of the model were unfavourable prognostic factors. The KM survival curve of the TCGA cohort revealed a lower survival rate in high‐risk patients, and this conclusion was validated in the ICGC cohort. We used ROC curves, calibration curves and decision curves to evaluate the predictive power of the nomogram. This reduces the error introduced by the prognostic risk model as a single prognostic indicator. In addition, we observed the prognostic role of traditional clinicopathological features in cancer, which is necessary from the molecular to the clinical level of application. Therefore, we analysed and visualized the differences in clinicopathological characteristics between risk subgroups, but the ICGC cohort only contains information in the stage column, so our analysis is limited in depth. We have also applied our model gene to the IMvigor210 immunotherapy cohort, and survival curve analysis suggests that high‐risk patients have more prolonged survival after immunotherapy. In a previous analysis, we found that the immune checkpoint gene expression was much higher in the high‐risk group than in the low‐risk group, demonstrating that our model can predict the effect of immunotherapy in patients. Unfortunately, the lack of expression of our target genes in the GEO database and the small number of samples did not allow us to validate our results further, and we will use more datasets to validate our findings in the future.

TRAF2 (TNF receptor‐associated factor 2) expression was found to be significantly increased in cancer tissues and was associated with tumour metastasis in previous studies,^22^ but has rarely been reported in hepatocellular carcinoma. By inhibiting apoptotic signalling, PGAM5 (PGAM family member 5) has been shown to be a poor prognostic factor for patients with hepatocellular carcinoma,^23^ which is consistent with our findings. ATG16L1 (autophagy related 16 like 1) was discovered to be an apoptotic molecule in HCC cells. J. iaranai Peantum reported that ATG16L1 protein was upregulated in tumour cell lines and promoted apoptosis in HepG2 cells.^24^ CARD9 (caspase recruitment domain family member 9) promotes metastasis‐associated macrophage polarisation, thereby promoting tumour metastasis. There is a high correlation between histopathological staging and metastasis of upregulated CARD9 expression.^25^ Alec E Vaezi et al.^26^ reported that PCYT1A (phosphate cytidylyltransferase 1A, choline) has biomarker value in patients with lung cancer and that high PCYT1A expression implies longer survival, but there is a lack of studies in hepatocellular carcinoma. The positive correlation between TLR2 (toll‐like receptor 2) expression and other proliferation and angiogenesis markers in hepatocellular carcinoma suggests a possible role for TLR2 in the pathogenesis of HCC.^27^ PARP2 (poly(ADP‐ribose) polymerase 2) is associated with different functions of cells in the innate immune response.^28^

According to the GSEA results, genes in the low‐risk group were enriched in the drug metabolism‐cytochrome P450 pathway, which is a key point of cancer treatment. They are involved in the inactivation and activation of anticancer drugs and mediate the metabolic activation of many procarcinogens.^29^ In addition, we performed sensitivity analyses of chemotherapeutic agents. The differential expression of our different prognostic genes influenced the effects of different agents. For example TRAF2 expression was positively correlated with the therapeutic effect of Cladribine, and Cladribine was reported to have an anticancer effect on human hepatocellular carcinoma HepG2 cells.^30^ Apigenin inhibits the expression of high‐risk genes and acts as an anticancer agent to induce apoptosis in hepatocellular carcinoma cells by inhibiting the P13K/Akt/mTOR pathway.^31^, ^32^ Conversely, Arecoline promotes the expression of high‐risk genes, and Arecoline is a carcinogen that enhances the risk of cancer in patients.^33^, ^34^ However, we cannot conclude that patients are necessarily sensitive to these drugs and more clinical trials are needed to verify this idea. While the high‐risk group had more immune cells, they also expressed more immune checkpoint genes, implying that the high‐risk group's immune function was more suppressed and that tumour cells had more opportunities to metastasize.

We further explored the mutations in the seven model genes and correlated them with the tumour mutation burden. We found that there were few mutations in the samples in our genes and few mutations in the structural domains of amino acids in seven of the genes. In addition, the correlation between risk score and TMB was not statistically significant. We encompassed previously published literature by extracting their model genes compared to our model. The survival of patients with hepatocellular carcinoma in the TCGA database was mainly located at 1–3 years. We used ROC curves and KM curves for analysis and comparison, and we found that our model was superior to other models in predicting the second‐year survival of patients.

Our study has some limitations. Experiments in vivo and in vitro are needed to confirm our findings and to do more in‐depth studies in the field of immunotherapy.

In conclusion, our study determined the prognostic and immunological roles of necroptosis‐related molecules such as TRAF2, PGAM5, ATG16L1, CADR9, PCYT1A, PARP2 and TLR2 in hepatocellular carcinoma. In addition, we developed a new predictive model for hepatocellular carcinoma to assess the efficacy of immunotherapy. This study identified novel biomarkers for hepatocellular carcinoma.

## CONFLICT OF INTEREST

The authors declare there are no conflicts of interest in this work.

## AUTHOR CONTRIBUTIONS

Y.H. contributed to the conceptualization of the study and the integrity of the study. Y.H. and Q.N.J., participated in the literature review and data collection. Y.H., and Q.N.J. participated in editing the manuscript and revising the manuscript. All authors read and approved the final manuscript.

## Supporting information

Figure S1

Appendix S1

Appendix S2

## Data Availability

The datasets used and/or analysed during the current study are available from the corresponding author on reasonable request.
