# Supplementary figures and images for: A multi‐omics‐based investigation of the immunological and prognostic impact of necroptosis‐related genes in patients with hepatocellular carcinoma
Source: J Clin Lab Anal. 2022 Mar 15;36(4):e24346. doi: 10.1002/jcla.24346 (PMC8993599; doi:10.1002/jcla.24346)

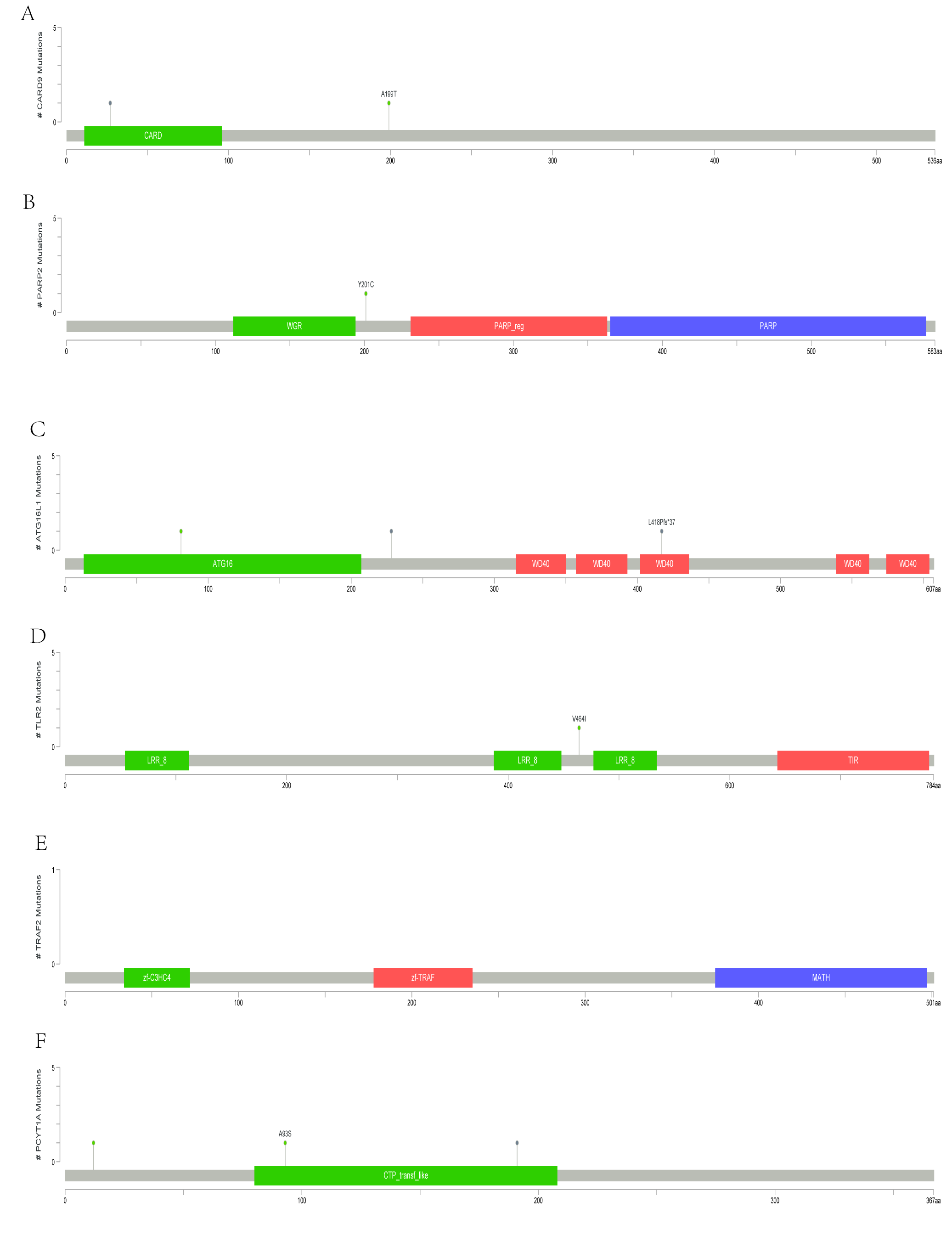

Supplement: Supplementary file 1 — Figure S1 [file JCLA-36-e24346-s003.tif]
